# Utility Evaluation Based on One-To-N Mapping in the Prisoner’s Dilemma Game for Interdependent Networks

**DOI:** 10.1371/journal.pone.0167083

**Published:** 2016-12-01

**Authors:** Juan Wang, Wenwen Lu, Lina Liu, Li Li, Chengyi Xia

**Affiliations:** 1 Tianjin Key Laboratory for Control Theory and Complicated Industry Systems, Tianjin University of Technology, Tianjin 300384, China; 2 School of Electrical Engineering, Tianjin University of Technology, Tianjin 300384, China; 3 Tianjin Key Laboratory of Intelligence Computing and Novel Software Technology, Tianjin University of Technology, Tianjin 300384, China; 4 Key Laboratory of Computer Vision and System (Ministry of Education), Tianjin University of Technology, Tianjin 300384, China; 5 School of Environmental Sciences, University of Guelph, Guelph, ON N1G2W1, Canada; Beihang University, CHINA

## Abstract

In the field of evolutionary game theory, network reciprocity has become an important means to promote the level of promotion within the population system. Recently, the interdependency provides a novel perspective to understand the widespread cooperation behavior in many real-world systems. In previous works, interdependency is often built from the direct or indirect connections between two networks through the one-to-one mapping mode. However, under many realistic scenarios, players may need much more information from many neighboring agents so as to make a more rational decision. Thus, beyond the one-to-one mapping mode, we investigate the cooperation behavior on two interdependent lattices, in which the utility evaluation of a focal player on one lattice may not only concern himself, but also integrate the payoff information of several corresponding players on the other lattice. Large quantities of simulations indicate that the cooperation can be substantially promoted when compared to the traditionally spatial lattices. The cluster formation and phase transition are also analyzed in order to explore the role of interdependent utility coupling in the collective cooperation. Current results are beneficial to deeply understand various mechanisms to foster the cooperation exhibited inside natural, social and engineering systems.

## Introduction

The persistence and emergency of cooperation is a widespread phenomenon in the nature and human being society, ranging from single cellular organisms to vertebrates, mammals, and even to populations [1, 2]. Yet, according to Darwinian evolutionary theory [3], any behavior that does not contribute to himself will eventually lead to the extinction of cooperation, which is rightly contradictory to many real-world situations. Therefore, deeply comprehending the persuasive cooperation conduct has become a challenging comission, and it is also a long-standing pendulum within the scientific communities [4]. To date, many researchers, who include the scientists coming from biology, physics, management and social sciences, often resort to the game theory to resolve the dilemma problem regarding the cooperation [5]. In particular, the evolutionary game theory builds a powerful framework to help to illustrate the evolution of cooperation [6], and some canonical mechanisms [7] have been identified as the effective means to enhance the collective cooperation level, such as kin selection [8], direct [9] or indirect reciprocity [10, 11], group selection [12] and so on.

In the recent years, a large plethora of studies are devoted to the explorations of evolutionary game on networks and graphs (see [13–15] for the comprehensive reviews) after the seminal discovery [16] in which the spatial structure, beyond the well-mixing topology, can make the cooperator survive well even in the most strict prisoner’s dilemma game (PDG) by organizing into the tightly cooperative clusters to defend the invasion of defectors. Meanwhile, the great progresses in the field of network science [17–21] further furnish a variety of topological structure for the games on complex graphs including small-world, scale-free, co-evolving and hierarchical ones. It has been found that the scale-free networks [22–26] might provide a unified platform to foster the cooperation for nearly all main social dilemmas, such as PDG, snowdrift game (SDG), public goods game (PGG) etc, and the evolutionary outcome is also very robust against the normalization of payoffs. Furthermore, the heterogeneity and diversity [27–35], which is considered by the individual behavior as well as those concerned in terms of players having different degree within a network, has been proved to be a very effective manner to promote the cooperation, and indeed many co-evolutionary rules [36–39] have also been introduced to spontaneously create the heterogeneous state so as to maintain the surprisingly high cooperation level (see [40] for a full review).

Very recently, however, it is clearly recognized that real-world systems are not isolated, but often interconnected or interdependent networks [41]. As an example, on the one hand, the communication systems (e.g., Mobile communication networks or Internet) are powered by the power grids; on the other hand, the operation and scheduling of power grids also rely on the communication systems, and their mutual dependency will lead to the extreme fragility even under random failures. Thus, the structure of and dynamics on interdependent networks receive a great deal of concern [42–45], and playing the games on multiple coupled networks also becomes an active topic in the realm of evolutionary game theory [46]. Among them, several methods are proposed to characterize the impact of coupled systems on the cooperation dynamics. One approach is to be considered in Refs. [47, 48] that the total networked population consists of two sub-populations (one layer of networks) including a different set of agents, in which they will play the games both with immediate neighbors on the same network and with those placed on the other one, the results reveal that the game types, density of interconnection links and topology of each sub-population will influence the final evolutionary results, even creating the polarized states. A second kind of work resolves the evolutionary dynamics between coupled networks via the multiplex modeling [49], where each agent can participate in multiple networks or layers and may possess different strategy states in different ones, meanwhile the individual strategy update at each layer is not based on the payoff accumulated only at that layer, but on the average payoff within all layers, and the authors find that, in the prisoner’s dilemma game, the cooperation is enhanced for a larger temptation parameter, but impaired at a smaller value for the temptation to defect. The third class of works devoting to the evolutionary games on coupled networks resort to the utility coupling or fitness evaluation between interdependent ones [50–56], in which the payoff calculation and update of the strategy for each agent will be confined at the same network, but the probability of strategy update will be correlated with the fitness evaluation integrating the payoffs obtained from the other network; Through this sort of utility coupling, the virtual connection between different networks is built and the role of interdependency in the promotion of cooperation is greatly impacted by the coupling way of utility or fitness assessment.

In previous works, the utility coupling is often implemented between one-to-one mapping agents on different networks, for example, Ref. [50] introduces the biased utility coupling for the PGG, in which the individual payoff on one network accounts for *α* and payoff of corresponding agent on the other networks occupies (1 − *α*), and finds that the aggregate level of cooperation on two networks is higher than that obtained on one isolated network; but for the PDG in Ref. [53], the utility function *U*_*x*_ is formulated as the linear combination of payoffs (*P*_*x*_ and *P*_*x*′_) of corresponding agents: *U*_*x*_ = *αP*_*x*_ + (1 − *α*)*P*_*x*′_, and the results indicate that there exists a critical interdependent factor *α*_*C*_, where the cooperation symmetry will be kept if *α* < *α*_*C*_ and spontaneous symmetry breaking of fraction of cooperators presents itself between different networks provided that *α* > *α*_*C*_. In Ref. [56], this kind of biased utility is not adopted, but an approach regarding the payoff superposition between different networks is applied, and it is unarguably uncovered that the cooperation phenomenon becomes richer and richer when compared to the cooperation behavior on single networks. In reality, the aforementioned works usually consider the utility computation only between corresponding players on distinct layers, that is, one-to-to mapping. However, under some real-world circumstances, much more information on the payoffs of multiple players from other networks will be beneficial for the focal one to make the decision. Thus, in this study, beyond the one-to-one mapping mode, we present an improved utility evaluation covering the combination of payoffs from corresponding partners on interdependent networks, in which the corresponding player and his nearest neighbors on the other network will be also taken into account when the player’s utility is calculated, that is, the game concerning a focal agent will be mapped into himself and multiple partners on the other network. The current work will further help to understand the role of interdependent network reciprocity in the evolution of cooperation within the social and natural systems.

## Methods and Materials

The Monte Carlo Simulation (MCS) is utilized to perform the evolution of cooperation behavior, and the system proceeds from two regular lattices with the linear size *L*, in which either lattice is satisfied with the conditions of periodic boundary. Initially, all players are allocated on the intersections of lattices, and designated as a cooperator (*s*_*x*_ = *C*) or a defector (*s*_*x*_ = *D*) with the equal probability (that is, being a cooperator or defector with the possibility of 50%). Thus, the system size or total number of players (*N*) is two times of the square of lattice linear size, *i.e.*, *N* = 2 × *L*^2^.

**Fig 1:**
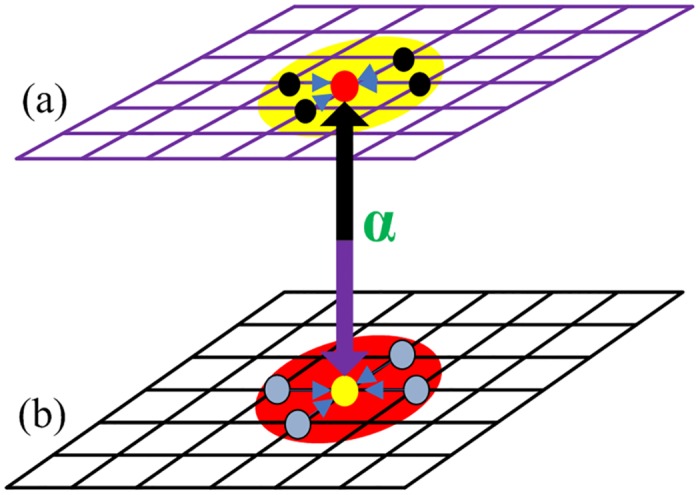
Illustration of one-to-N mapping utility evaluation for interdependent networks. In this model, payoff obtaining and strategy transfer can only take place between players on the same network. The system consists of two *L* × *L* lattices [panel (a) and panel (b)], and the utility or fitness evaluation of the focal agent will be correlated with her own payoff and those of corresponding partners on the other network. As an example, the focal player [red circle in panel (a) or yellow circle in panel (b)] will be coupled with those in the other network [the red-shaded ones in panel (b) or yellow-shaded ones in panel (a)].

After that, starting from a randomly chosen player from any network, the pairwise interaction and payoff computing will be performed according to the prisoner’s dilemma as follows

$$\begin{array}{c} \begin{array}{cc} \begin{array}{cc} \;\;C & \;D \end{array} & \\ \begin{array}{c} C\\ D \end{array}(\begin{array}{cc} R & \;S\\ T & \;P \end{array}) \end{array} \end{array}$$ (1)

where *R* and *P* denote the payoff of reward or punishment when two players take the same action: cooperation or defection; and the cooperator gets the sucker’s payoff (*S*), but the defector cannot resist the temptation and obtain the highest payoff when they choose the different strategy; Meanwhile, *T* > *R* > *P* > *S* and 2*R* > *S* + *T* must be satisfied for the standard PDG model. Since *T* > *R* and *P* > *S*, it is obvious that to defect is the best option for a player regardless of his opponent’s choice, that is, defection becomes the Nash equilibrium of PDG. Without loss of generality, we adopt a class of weak PDG parameter setup: 1 < *T* = *b* < 2, *R* = 1 and *P* = *S* = 0, which is firstly applied by Nowak & May [16] and nearly captures all PDG’s features during the strategy game. Henceforth, each player (say *x*) can calculate his or her payoff (Π_*x*_) by playing with 4 nearest neighbors as we can only discuss the typical von-Neumann neighborhood setup in this study. Then, the individual utility or fitness will be assessed for the evolutionary iteration, and the focal agent’s utility evaluation will not only be related with his own payoff, but also with the average of payoff of players from the other lattice, which include the corresponding partner (say *x*′) and his 4 immediate neighbors. Among them, player *x*′ and his neighbors will also apply the identical method to compute their payoffs according to Eq (1), and their average value $\bar{P}$ will be calculated as follows,

$$\begin{array}{c} \bar{\Pi }=\frac{\Pi_{x^{\prime }}+\sum_{i^{\prime }\in N_{x^{\prime }}}\Pi_{i^{\prime }}}{5} \end{array}$$ (2)

where *N*_*x*′_ denotes the von-Neumann neighborhood of player *x*′ and *i*′ represents one of members in the neighborhood (*i.e.*, one of nearest neighbors of player *x*′). It is worth mentioning that each agent’s payoff can be only obtained by playing with 4 nearest neighbors on the same lattice, not be allowed to directly integrate the interaction between individuals on different lattices. Thus, we consider the focal player’s utility as a linear combination of Π_*x*_ and $\bar{\Pi }$ in the following way,

$$\begin{array}{c} U_{x}=\Pi_{x}+\alpha \bar{\Pi } \end{array}$$ (3)

where *α* is a tunable parameter which means the extent of average payoff integration into the current utility assessment, without loss of generality, *α* usually lies between 0 and 1. Different from previous works [50–56], the utility computation will cover the focal one and multiple partners on the other network as shown in Fig 1, that is, beyond the usual one-to-one mapping assumption for the evolution of cooperation on interdependent networks.

Next, the focal player will try to update his current strategy by comparing the utility between himself (say *x*) and one of nearest neighbors picked up at random (say *y*), in which player *y* will acquire his utility in the same method with the focal one according to the above-mentioned procedure. The strategy (*s*_*x*_) of player *x* will be changed into the strategy (*s*_*y*_) of player *y* with the following probability

$$\begin{array}{c} W_{s_{x}\leftarrow s_{y}}=\frac{1}{1+e^{\frac{U_{x}-U_{y}}{\kappa }}} \end{array}$$ (4)

where *κ* stands for the uncertainty of strategy adoption [57] or the individual irrationality for an agent to make a decision (*κ* is usually set to be 0.1 unless stated before), *U*_*x*_ and *U*_*y*_ denote the utilities of players *x* and *y*, respectively.

Finally, one full MCS step will be completed provided that each agent will have a chance to update his strategy on average. In the following section, the lattice configuration is set to be 2 × 200 × 200, and larger lattice size (*e.g.*, *L* = 300 or *L* = 400) is also tested and qualitatively similar results are obtained. Moreover, all numerical simulation results will be averaged over 20 independent runs for the same parameter setup.

## Results and Analysis

**Fig 2:**
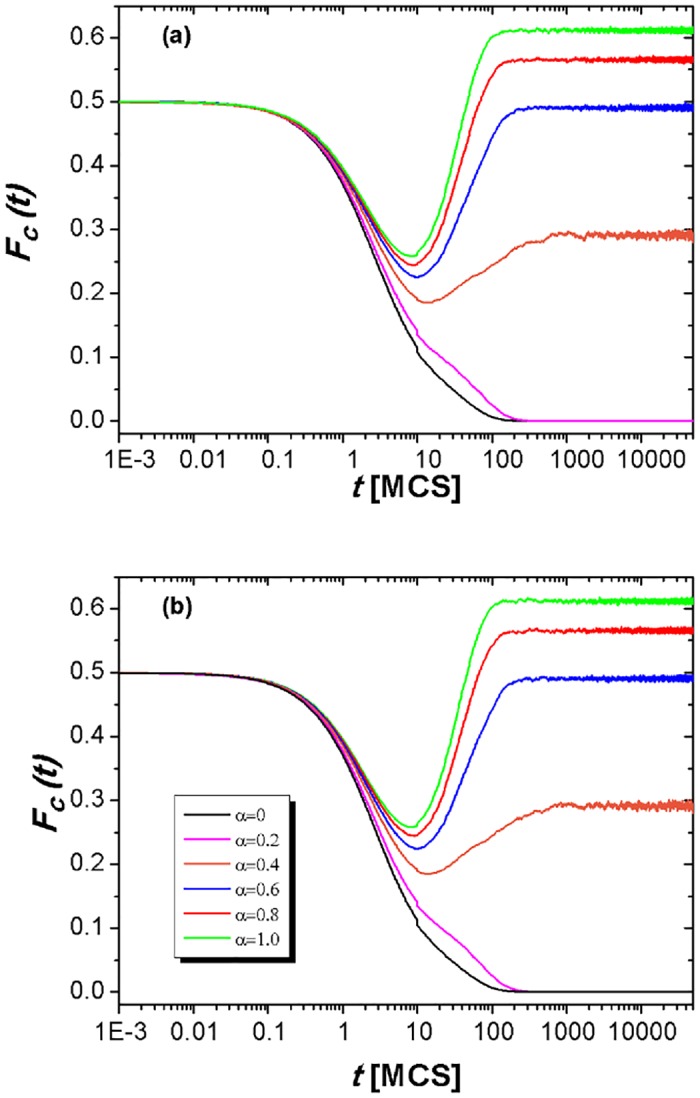
Fraction of cooperators [*F*_*C*_(*t*)] as a function of MCS time step for a fixed temptation to defect (*b* = 1.07). Panel (a) and (b) characterize the evolution of cooperation on two independent lattices, respectively. The system setup is assumed to be *L* = 200, *MCS* = 5 × 10^4^ and *κ* = 0.1, and the tunable parameter *α* is set to be 0, 0.2, 0.4, 0.6, 0.8 and 1.0.

**Fig 3:**
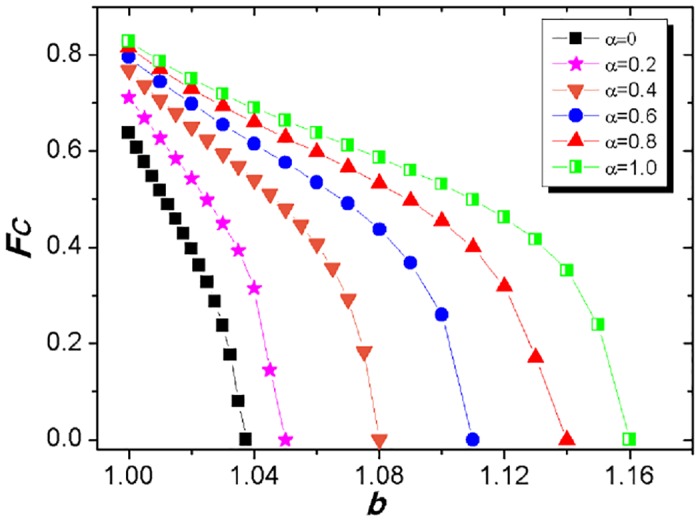
Fraction of cooperators (*F*_*C*_) at the stationary state as a function of temptation to defect (*b*). The system setup is assumed to be *L* = 200, *MCS* = 5 × 10^4^ and *κ* = 0.1, and the tunable parameter *α* is set to be 0, 0.2, 0.4, 0.6, 0.8 and 1.0, respectively.

Firstly, in order to deeply explore the role of utility coupling, we provide the evolution of fraction of cooperators at each time step for a fixed defection parameter (*b* = 1.07) in Fig 2, where the tunable parameter (*α*) can be set as 0, 0.2, 0.4, 0.6, 0.8 and 1.0, respectively. On the one hand, the simulation results convincingly turned out that the cooperation behavior can be greatly improved on account of the introduction of utility coupling; On the other hand, we can observe that the behavior of *F*_*C*_(*t*) inside two panels [panel (a) and (b)] is fully identical, that is, the cooperation symmetry always holds for various tunable parameters *α*. Moreover, for the first 10 time steps, we divide one MCS time step into 1000 sub-steps so that the evolutionary process of *F*_*C*_(*t*) at each time step can be carefully scrutinized. As we can see, the time course of cooperation can be divided into two evident phases: the enduring (END) period and the expanding (EXP) period. Initially, in the END period, the fraction of cooperators will gradually reduced regardless of whether the utility coupling exists, which shows that the defectors will dominate the population since the Nash equilibrium of PDG is the defection. However, after several steps, the cooperation exhibits a small bit different scenario and the EXP period emerges. From this period, the cooperators begin to win the superiority over the defectors for the utility coupling takes effect. Among them, *α* = 0 or *α* = 0.2, the eventual fate of the whole population is the full defection, which shows that only the spatial reciprocity or the very weak utility coupling is not enough to support the evolution of cooperation. However, *α* becomes larger and larger, for example, *α* is set to be equal to or higher than 0.4, the fraction of cooperators can be finally maintained at a stable level since the cooperators can organize into compact clusters to resist the invasion of defectors (which will be further illustrated in the following paragraph). In the meantime, the larger the tunable parameter *α*, the higher the stationary fraction of cooperators, and the current results are also consistent with those in Fig 3. Thus, the utility coupling between two interdependent lattices has altered the behavior of cooperation by a large margin when compared to the traditionally spatial lattice setup (i.e., *α* = 0).

Next, since the symmetry for the fraction of cooperators can be well kept on two panels under various *α*, we will record the average value of cooperator’s density at the stationary state on two lattices as the fraction of cooperators (*F*_*C*_) among the whole population. Here, we depict *F*_*C*_ as a function of temptation to defect (*b*) in Fig 3 for various tunable parameters to check the role of utility coupling under the one-to-N mapping condition. It can be clearly observed that the level of cooperation can be generally promoted with the introduction of utility coupling from the corresponding partners on the other network. Among them, when *α* is equal to 0, the focal player’s utility calculation does not refer to any other partner, the current model will be equivalent to the traditional PDG model and our simulation results are also identical with previous works. However, when *α* > 0, the focal player computes his utility by considering the average value of corresponding partners through the 1-to-*N* mapping, and the numerical simulations unamgiguously indicate that *F*_*C*_ can be elevated as *α* increases. Meanwhile, the larger the adjustable parameter *α*, the higher the fraction of cooperators *F*_*C*_. As an example, *α* = 0., the fraction of cooperators still arrives at around 60% when *b* = 1.0375 where the cooperation becomes extinct in the standard PDG, the maximum defection parameter (*b*_*m*_) leading to the extinction of cooperation arrives at *b* = 1.08; Of particular note is that the extinction threshold *b*_*m*_ can be up to 1.16, which is often extra-ordinarily high as compared to the standard spatial PDG. From this point, only the spatial reciprocity can be not enough to create this kind of favorable situations to foster the cooperative individuals. Accordingly, the exterior information obtained from the other network can be added into the current utility evaluation, the focal agent will become much more rational for the strategy choice since he can hold the richer information to aid his decision making. Taking together, *F*_*C*_ becomes higher and higher as the tunable parameter *α* increases under the identical defection parameter *b* since the novel utility coupling has been added into the current model.

**Fig 4:**
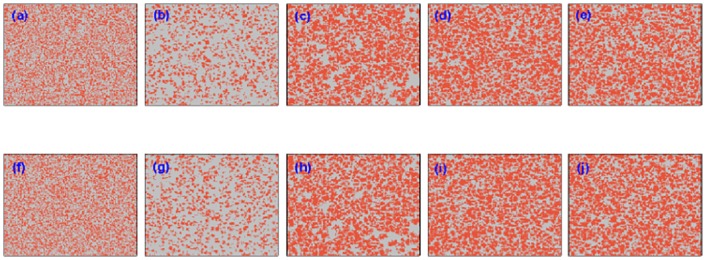
Characteristic snapshots of cooperators and defectors on two independent lattices for a fixed temptation to defect (*b* = 1.07) starting from an initial strategy distribution. In the upper row, from panel (a) to (e), snapshots are taken from one lattice at the time step 1, 10, 100, 1000, 50000, respectively; Similarly, in the lower row, we provide the snapshots on the other lattice at the same time step. The orange dots represent the cooperators, and the light gray dots denote the defectors. The system setup is assumed to be *L* = 200, *MCS* = 5 × 10^4^ and *κ* = 0.1 and the tunable parameter *α* = 0.8.

**Fig 5:**
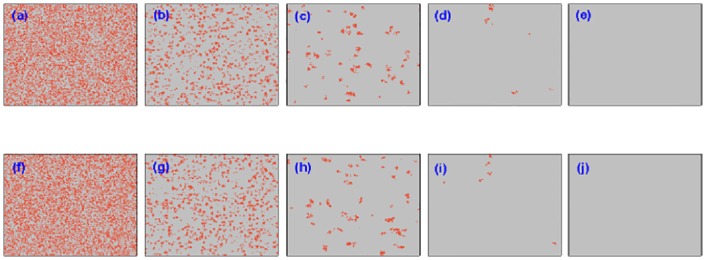
Characteristic snapshots of cooperators and defectors on two independent lattices for a fixed temptation to defect (*b* = 1.07) starting from an initial strategy distribution. In the upper row, from panel (a) to (e), snapshots are taken from one lattice at the time step 1, 10, 100, 200, 1000, respectively; Similarly, in the lower row, we provide the snapshots on the other lattice at the same time step. The orange dots represent the cooperators, and the light gray dots denote the defectors. The system setup is assumed to be *L* = 200, *MCS* = 5 × 10^4^ and *κ* = 0.1 and the tunable parameter *α* = 0.2.

Regarding the competition properties between cooperators and defectors during the evolution of system status, how the cooperators accumulated the advantage over the defectors is still to be resolved. We make a preliminary attempt to check the formation of cooperative clusters by providing the snapshots at several different time steps for a specified tunable parameter *α* = 0.8 in Fig 4. Here, the upper row panels characterize the snapshots on one lattice, and from left [panel (a)] to right [panel (e)], the time step is set to be *MCS* = 0, 10, 100, 1000, 50000, respectively. It can be shown that the strategy of agents is randomly assigned at the initial time [panel (a)], hence the cooperators or defectors nearly occupy the equal space and they cannot be discerned visually. After 9 steps, the defectors obtained the superior position during the game playing and the light gray dots obviously occupy most of space in panel (b). However, some compact and cooperative clusters are sporadically organized so that the orange dots still exist within the population [panel (b)]. In the following steps [from panel (c) to (e)], the cooperators gradually accumulate the evolutionary advantage over defectors during the competition between them and eventually arrive at the stationary state. Accordingly, the corresponding snapshots on the lower panels depict the distribution of cooperators and defectors on the other lattice, and the identical evolving tendency can be observed. Furthermore, the similar pattern analysis is performed for another tunable parameter *α* = 0.2 in Fig 5, where the snapshots are taken at *MCS* = 0, 10, 100, 200 and 1000 (since the cooperators are fully extinct after 1000 steps), and it can be examined that the cooperators will lose the superiority during the process of game playing since the extent of utility coupling from the other lattice is not strong enough. At the same time, the cooperation symmetry can be kept substantially well between the corresponding upper and lower panels, which is also consistent with results displayed in Fig 2.

**Fig 6:**
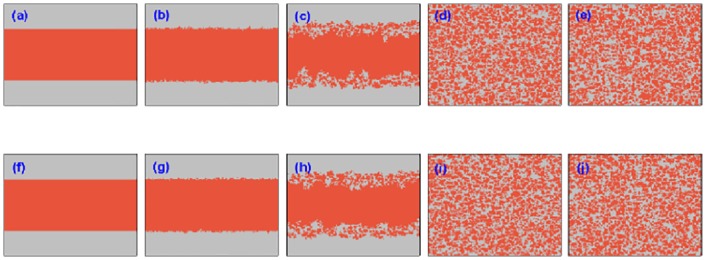
Characteristic snapshots of cooperators and defectors on two independent lattices for a fixed temptation to defect (*b* = 1.07) starting from a prepared strategy distribution. In the upper row, from panel (a) to (e), snapshots are taken from one lattice at the time step 1, 10, 100, 1000, 50000, respectively; Similarly, in the lower row, we provide the snapshots on the other lattice at the same time step. The orange dots represent the cooperators, and the light gray dots denote the defectors. The system setup is assumed to be *L* = 200, *MCS* = 5 × 10^4^ and *κ* = 0.1 and the tunable parameter *α* = 0.8.

**Fig 7:**
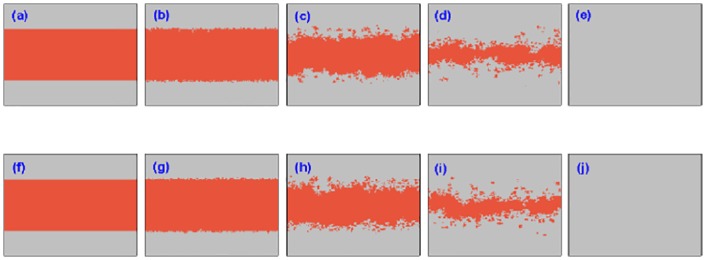
Characteristic snapshots of cooperators and defectors on two independent lattices for a fixed temptation to defect (*b* = 1.07) starting from a prepared strategy distribution. In the upper row, from panel (a) to (e), snapshots are taken from one lattice at the time step 1, 10, 100, 1000, 50000, respectively; Similarly, in the lower row, we provide the snapshots on the other lattice at the same time step. The orange dots represent the cooperators, and the light gray dots denote the defectors. The system setup is assumed to be *L* = 200, *MCS* = 5 × 10^4^, *κ* = 0.1 and the tunable parameter *α* = 0.2.

To further account for the origin favoring the cooperation in the current PDG model, Figs 6 and 7 depict the evolution of characteristic snapshots for two different utility coupling strength *α* = 0.8 and *α* = 0.2, where the initial cooperators are preset in the middle of lattice and defectors are evenly distributed on both sides of lattice. In Fig 6, the coupling strength *α* is set to be 0.8, and all other simulation parameters are also wholly same as those in Fig 4. Two rows denote the setup of two-layered lattices, and 5 columns characterize the snapshots at different *MCS* time steps which are, from left to right, *MCS* = 0, 10, 100, 1000, 50000, respectively. Although the initial distribution of cooperators and defectors is utterly different from that in Fig 4, the evolutionary process is analogous, and cooperators can gradually organize into the compact clusters with the help of utility evaluation borrowing from the payoffs of players on the other lattice, to defend from the exploitation of defectors and lead to the situation in which two strategies can coexist at the stationary state. Henceforth, the final evolutionary outcome is identical with the case of randomly initial deployment. Similarly, in Fig 7, *α* = 0.2 is not enough to support the persistence of cooperation and free-riding from defectors thrives so that the cooperators cannot resist the invasion of defectors and the cooperators are finally extinct, the evolution of snapshot is also akin to Fig 5 where the snapshots are taken at *MCS* = 0, 10, 100, 200 and 1000 (from left to right). In addition, the evolutionary process still maintains the symmetry of cooperation between two lattices. Taking together, the collective cooperation evolution in the whole population is totally independent of their initial setup on two lattices, and the strength of utility coupling determines their final fate.

**Fig 8:**
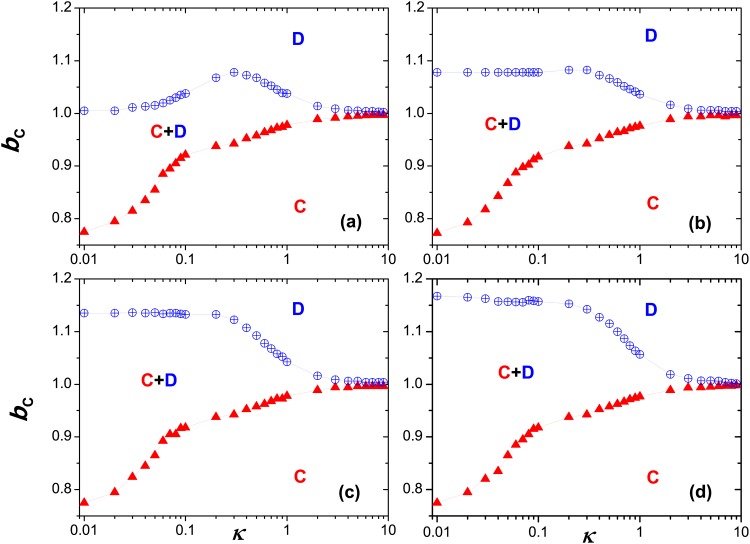
Full *b* − *κ* phase diagram. From panel (a) to (d), the tunable parameter is set to be *α* = 0, 0.4, 0.8 and 1.0, respectively. Here, the yellow crossed circle denotes the transition between *C* + *D* (coexistence phase) and *D* (full defection), and the read upper triangle represents the transition between *C* + *D* and *C* (full cooperation). The system setup is assumed to be *L* = 200, *MCS* = 5 × 10^4^.

The impact of utility coupling between two interdependent lattices can be examined in depth by checking the full *b* − *κ* phase diagram in Fig 8, which fully demonstrates the robustness of cooperation against the noise strength (i.e., the uncertainty of strategy selection *κ*), unlike some previous works where the noise may substantially vary the collective cooperation ratio with the whole population [58]. In Fig 8, and from panel (a) to (d), the tunable parameter *α* is set to be 0, 0.4, 0.8 and 1.0, respectively, at the same time the pure cooperating (defecting) region is denoted by the capital *C* (*D*), while *C* + *D* characterizes the coexistence region containing cooperators and defectors within systems, and the upper triangle (crossed circle) symbol means the critical value standing for the transition from pure cooperators to mixed cooperators and defectors (mixed ones to pure defectors). From Fig 8, the general trend is that, as *α* increases, the pure defecting regions is greatly compressed, and the mixing or coexistence regions is largely broadened, but the pure cooperating regions is almost kept to be unchanged. Among them, in panel (a), *α* is equal to 0, which implies that the system is reduced to two traditional and independent lattice systems and our results reproduce the classical bell-shaped ones regarding the phase transition, and current phase diagrams are consistent with many previous results. However, when *α* is greater than 0, and the utility evaluation need to consider the impact of corresponding partners on the other lattice, henceforth render that the phase diagram differs a little from the classical ones and the bell-shaped curve indicating the transition between *D* and *C* + *D* disappears, which can be observed from panel (b) to (d). Meanwhile, as *κ* lies between 0.01 and 1.0, it is easier for the cooperators and defectors to coexist; while *κ* > 1.0, the range of temptation *b* for them to coexist becomes very narrow, and it is particular for *κ* > 3.0, the coexistence between cooperators and defectors becomes impossible, that is, the system could only get into the full defection or cooperation status as *b* varies. Since the very large noise strength (*κ*) leads to the fact that the probability to select the cooperation or defection is almost not influenced by the utility difference and the system tends to trap into one of two states, full cooperation or complete defection. Taking together, introducing the payoff coupling into the individual utility evaluation will greatly change the behaviors of phase transition between the cooperation and defection on the square lattice.

## Conclusions and Discussion

In summary, we discuss the evolution of cooperation behavior on two interdependent lattices through the utility coupling between corresponding players. In many previous works, this type of utility coupling is often implemented with one-to-one mode, that is, the evaluation of one player’s utility on one lattice is only correlated with his own payoff and one corresponding partner’s payoff on the other lattice. In reality, the individual decision may be dependent on many factors including his own or other individual’s payoffs. Thus, in this paper, beyond one-to-one pattern, we propose a novel utility coupling way based on himself and several corresponding players on the other networks, that is, one-to-N mapping mode, and the numerical simulation results indicate that the cooperation behavior on the spatial lattices can be largely modified. Firstly, the total level of collective cooperation at the stationary state can be greatly promoted as the extent of payoff integration into the individual utility evaluation (*i.e.*, tunable parameter *α*) increases, which means that the larger the tunable parameter *α*, the higher the level of stationary state. Secondly, the coupling of utility assessment concerning multiple agents on the other lattice is beneficial for the focal player to make a rational choice and helps to create the cooperative clusters to defend the invasion of defectors. Lastly, this novel utility coupling also alters the properties regarding the phase transition when compared to the classical spatial lattices. The current results further enrich the understanding of widespread cooperation phenomena within many natural, social and even engineering systems, especially aiding to illustrate the role of interdependency and network reciprocity in the promotion of cooperation.

The future works will extend the current one-to-N mapping mode into other spatial game models (e.g., snowdrift game and public goods game) or other heterogenous topologies (i.e., interdependent random graphs and scale free networks), to further explore whether the cooperation level can be enhanced within these scenarios by the one-to-N mapping mode. Furthermore, the evolution of cooperation within three or more networks can also deeply investigated since many real-world systems are often interwoven through multiple subsystems.

## References

[1] Coakley S, Nowak MA, eds (2013). Evolution, Games and God: The Principle of Cooperation. Cambrige, MA: Harvard University Press 10.1007/s12232-013-0184-2

[2] Axelrod R (2006) The Evolution of Cooperation (Basic Books; New York).

[3] Darwin C (1859) The Origin of Species. Cambrige, MA: Harward University Press (Reprinted, 1964).

[4] Maynard Smith J (1982) Evolution and the Theory of Games. Cambridge, UK: Cambridge University.

[5] Gintis H (2000) Game Theory Evolving. Princeton, NJ: Princeton University Press.

[6] Nowak MA (2006) Evolutionary Dynamics: Exploring the equations of life. Cambrige, MA: Harvard Universtiy Press.

[7] Nowak MA (2006) Five rules for the evolution of cooperation. Science 314: 1560–1563. 10.1126/science.1133755 17158317 PMC3279745

[8] Hamilton WD (1964) The genetical evolution of social behaviour. J Theor Bio 7: 1–52. 10.1016/0022-5193(64)90038-4 5875341

[9] Trivers RL (1971) The evolution of reciprocal altruism. Q Rev Biol 46: 35–37. 10.1086/406755

[10] Nowak MA and Sigmund K (1998) Evolution of indirect reciprocity by image scoring. Nature 393: 573–537. 10.1038/31225 9634232

[11] Panchanathan K, Boyd R (2004) Indirect reciprocity can stabilize cooperation without the second-order free rider problem. Nature 432: 499–502. 10.1038/nature02978 15565153

[12] Traulsen A, Nowak MA (2006) Evolution of cooperation by multilevel selection. Proc Natl Acad Sci(USA) 103: 10952–10955. 10.1073/pnas.0602530103 16829575 PMC1544155

[13] Szabó G, Fáth G (2007). Evolutionary games on graphs. Phys Rep 446: 97–216. 10.1016/j.physrep.2007.04.004

[14] Roca CP, Cuesta JA, Sánchez A (2009) Evolutionary game theory: Temporal and spatial effects beyond replicator dynamics. Phys Life Rev 6: 208–249. 10.1016/j.plrev.2009.08.001 20416850

[15] Perc M, Gómez-Gardeñes J, Szolnoki A, Floría LM and Moreno Y (2013) Evolutionary dynamics of group interactions on structured populations: A review. J R Soc Interface 10: 20120997 10.1098/rsif.2012.0997 23303223 PMC3565747

[16] Nowak MA, May RM. Evolutionary games and spatial chaos. Nature 1992; 359: 826–829. 10.1038/359826a0

[17] Boccaletti S, Latora V, Moreno Y, Chavezf M, Hwang DU (2006) Complex networks: structure and dynamics. Phys Rep 424: 175–308. 10.1016/j.physrep.2005.10.009

[18] Abramson G, Kuperman M (2001) Social games in a social network. Phys Rev E 63:030901(R) 10.1103/PhysRevE.63.030901 11308622

[19] Tomassini M, Luthi L, Giacobini M (2006) Hawks and doves on small-world networks. Phys Rev E 73: 016132 10.1103/PhysRevE.73.016132 16486241

[20] Gómez-Gardeñ es J, Campillo M, Moreno Y, Floría LM (2007) Dynamical organization of cooperation in complex networks. Phys Rev Lett 98: 108103 10.1103/PhysRevLett.98.108103 17358570

[21] Xia CY, Meloni S, Perc M, Moreno Y (2015) Dynamic instability of cooperation due to diverse activity patterns in evolutionary social dilemmas. EPL 109: 58002 10.1209/0295-5075/109/58002

[22] Santos FC, Pacheco JM (2005) Scale-free networks provide a unifying framework for the emergence of cooperation. Phys Rev Lett 95: 098104 10.1103/PhysRevLett.95.098104 16197256

[23] Doebeili M, Hauert C (2005) Models of cooperation based on the prisonser’s dilemma and the snowdrift game. Ecol Lett 8: 748–766. 10.1111/j.1461-0248.2005.00773.x

[24] Santos FC, Santos MD, Pacheco JM (2008) Social diversity promotes the emergence of cooperation in public goods games. Nature 454: 213–216. 10.1038/nature06940 18615084

[25] Du WB, Cao XB, Hu MB, Wang WX (2009) Asymmetric cost in snowdrift game on scale-free networks, EPL 87: 60004 10.1209/0295-5075/87/60004

[26] Du WB, Cao XB, Zhao L, Hu MB (2009) Evolutionary games on scale-free networks with a preferential selection mechanism. Physica A 388: 4509–4514. 10.1016/j.physa.2009.07.012

[27] Chen MH, Wang L, Sun SW, Wang J, Xia CY (2016) Evolution of cooperation in the spatial public goods game with adaptive reputation assortment. Phys Lett A 380: 40–47. 10.1016/j.physleta.2015.09.047

[28] Wu ZX, Xu XJ, Wang YH (2006) Prisoner’s dilemma game with heterogeneous influental effect on regular small-world networks. Chin Phys Lett 23: 531–534. 10.1088/0256-307X/23/3/002

[29] Fort H (2008) A minimal model for the evolution of cooperation through evolving heterogenous games. EPL 81: 48008 10.1209/0295-5075/81/48008

[30] Masuda N (2008) Oscillatory dynamics in evolutionary games are suppressed by heterogeneous adaption rates of players. J Theor Biol 251: 181–189. 10.1016/j.jtbi.2007.11.010 18086478

[31] Perc M, Szolnoki A (2008) Social diversity and promotion of cooperation in the spatial prisoner’s dilemma game. Phys Rev E 77: 011904 10.1103/PhysRevE.77.011904 18351873

[32] Droz M, Szwabiński J, Szabó G (2009) Motion of influential players can support cooperation in prisoner’s dilemma. Eur Phys J B 71: 579–585. 10.1140/epjb/e2009-00160-1

[33] Zhu CJ, Sun SW, Wang L, Ding S, Wang J, Xia CY (2014) Promotion of cooperation due to diversity of players in the spatial public goods game with increasing neighborhood size. Physic A 406: 145–154. 10.1016/j.physa.2014.03.035

[34] Wang Z, Wang L, Yin ZY, Xia CY (2012) Inferring reputation promotes the evolution of cooperation in spatial social dilemma games. PloS one 7: e40218; 10.1371/journal.pone.0040218 22808120 PMC3392274

[35] Zhang Y, Wang L, Zhang YQ, Li X (2012) Towards a temporal network analysis of interactive WiFi users. EPL 98: 68002 10.1209/0295-5075/98/68002

[36] Ebel H, Bornholdt S (2002) Coevolutionary games on networks. Phys Rev E 66: 056118 10.1103/PhysRevE.66.056118 12513567

[37] Perc M, Szolnoki A (2008) Coevolution of teaching activity promotes cooperation. New J Phys 10: 043036 10.1088/1367-2630/10/4/043036

[38] Chen MH, Wang L, Wang J, Sun SW, Xia CY (2015) Impact of individual response strategy on the spatial public goods game within mobile agents. Applied Mathematics and Computation, 251: 192–202. 10.1016/j.amc.2014.11.052

[39] Zimmermann MG, Eguíluz VM, San Miguel M (2004) Coevolution of dynamical states and interactions in dynamic networks. Phys Rev E 69: 065102(R) 10.1103/PhysRevE.69.065102 15244650

[40] Perc M, Szolnoki A (2010) Coevolutionary games-A mini review. BioSystems 99: 109–125. 10.1016/j.biosystems.2009.10.003 19837129

[41] Buldyrev SV, Parshani R, Paul G, Stanley HE, Havlin S (2010) Catastrophic cascade of failures in interdependent networks. Nature 464: 1025–1028. 10.1038/nature08932 20393559

[42] Boccaletti S, Bianconi G, Criado R, Genio C-I, Gómez-Gardeñs J, Romance M, Sendiña-Nadal I, Wang Z, Zanin M (2014) The structure and dynamics of multiplayer networks. Phys Rep 544: 1–122. 10.1016/j.physrep.2014.07.001 PMC7332224 32834429

[43] Kivelä M, Arenas A, Barthelemy M, Gleeson LP, Moreno Y (2014) Multilayer networks. J Complex Networks 2: 203–271. 10.1093/comnet/cnu016

[44] Gao J, Buldyrev SV, Stanley HE, Havlin S (2012) Networks formed from interdependent networks. Nat Phys 8: 40–48. 10.1038/nphys2180 23005189

[45] Wang L, Li X (2014) Spatial epidemiology of networked metapopulation: An overview. Chin Sci Bull, 59: 3511–3522. 10.1007/s11434-014-0499-8 PMC7088704 32214746

[46] Wang Z, Wang L, Szolnoki A, Perc M (2015) Evolutionary games on multiplayer networks: A colloquim, Euro Phys J B 88: 1–15. 10.1140/epjb/e2015-60270-7

[47] Gómez-Gardeñs J, Gracia-Lázaro C, Floría LM, Moreno Y (2012) Evolutionary dynamics on interdependent populations. Phys Rev E 86: 056113 10.1103/PhysRevE.86.056113 23214849

[48] Jiang LL, Perc M (2013) Spreading of cooperative behaviour across interdependent groups. Sci Rep 3: 2483 10.1038/srep02483 23963495 PMC3748424

[49] Gómez-Gardeñs J, Reinares I, Arenas A, Floria M (2012) Evolution of cooperation in multiplex networks, Sci. Rep. 2: 620 10.1038/srep00620 22943006 PMC3431544

[50] Wang Z, Szolnoki A, Perc M (2012) Evolution of public cooperation on interdependent networks: The impact of biased utility functions. EPL 97: 48001 10.1209/0295-5075/97/48001

[51] Wang Z, Szolnoki A, Perc M (2013) Optimal interdependence between networks for the evolution of cooperation. Sci Rep 3: 2470 10.1038/srep02470 23959086 PMC3747507

[52] Wang Z, Wang L, Perc M (2014) Degree mixing in multilayer networks impedes the evolution of cooperation. Phys Rev E 89: 052813 10.1103/PhysRevE.89.052813 25353850

[53] Jin Q, Wang L, Xia CY, Wang Z (2014) Spontaneous symmetry breaking in interdependent networked game. Sci Rep 4: 4095 10.1038/srep04095 24526076 PMC3924213

[54] Xia CY, Miao Q, Wang J, Ding S (2014). Evolution of cooperation in the traveler’s dilemma game on two coupled lattices. Applied Mathematics and Computation 246: 389–398. 10.1016/j.amc.2014.08.006

[55] Meng XK, Xia CY, Gao ZK, Wang L, Sun SW (2015) Spatial prisoner’s dilemma games with increasing neighborhood size and individual diversity on two interdepdendent lattics. Phys Lett A 379: 767–773. 10.1016/j.physleta.2014.12.051

[56] Xia CY, Meng XK, Wang Z (2015) Heterogeneous coupling between interdependent lattices promotes the cooperation in the prisoner’s dilemma game. PLoS ONE 10: e0129542 10.1371/journal.pone.0129542 26102082 PMC4477883

[57] Szabó G, Töke C (1998) Evolutionary prisoner’s dilemma game on a square lattice. Phys Rev E 58: 69–73. 10.1103/PhysRevE.58.69

[58] Zhang GQ, Sun QB, Wang L (2013) Noise induced enhancement of network reciprocity in social dilemmas. Chaos Solitons and Fractals 51: 31–35. 10.1016/j.chaos.2013.03.003

